# Recommendations for the Development of Telemedicine in Poland Based on the Analysis of Barriers and Selected Telemedicine Solutions

**DOI:** 10.3390/ijerph19031221

**Published:** 2022-01-22

**Authors:** Kamila Furlepa, Anna Tenderenda, Remigiusz Kozłowski, Michał Marczak, Waldemar Wierzba, Andrzej Śliwczyński

**Affiliations:** 1 Satellite Campus in Warsaw, University of Humanities and Economics in Lodz, 90-212 Lodz, Poland; kamila.furlepa@gmail.com (K.F.); wwierzba@post.pl (W.W.); 2 Department of Hygiene and Health Promotion, Medical University of Lodz, 90-419 Lodz, Poland; anna.tenderenda@stud.umed.lodz.pl; 3 Centre for Security Technologies in Logistics, Faculty of Management, University of Lodz, 90-237 Lodz, Poland; remigiusz.kozlowski@wz.uni.lodz.pl; 4 Department of Management and Logistics in Healthcare, Medical University of Lodz, 90-131 Lodz, Poland; michal.marczak@umed.lodz.pl

**Keywords:** telemedicine, e-health, telemedicine barriers, development of telemedicine, telemedicine solutions

## Abstract

Technological development around the world has led to the digitalisation of the health system. Along with the digitalisation of the health sector, financial, legal, awareness-related, technological and IT barriers appeared. The aim of the article is to present recommendations for the development of telemedicine services in Poland on the basis of a list of implementation barriers and ways of resolving them in the USA and selected European countries. A literature review was conducted in accordance with the PRISMA-ScR, using the PubMed and Google Scholar databases, Scopus and the OECD iLibrary. A total of 59 literature positions were used, which constituted the references. The article presented the implemented and effective solutions in selected countries. Based on these solutions, recommendations for the development of telemedicine in Poland were presented, as well as successes in the form of telemedicine startups, which can inspire other countries. The analysis of the publications discussed in the article shows that the implementation of telemedicine services should begin with the elimination of barriers limiting the development of telemedicine systems. An important issue in their elimination is to analyse their interconnections and implement such solutions which would have a multi-area coverage.

## 1. Introduction

One of the fastest growing branches related to medicine is the segment that uses the achievements of modern computer science, known as telemedicine. We owe this dynamic development to new technologies that support the work of medical personnel and allow for a quick and accurate diagnosis. The experience resulting from the introduction of telemedicine services in the world has been the successful reduction in time and geographical barriers in traditional methods of providing medical services. With the advancement of telemedicine, however, new barriers have emerged that need attention so that we could widely use the achievements of telemedicine.

The development of systems enabling diagnosing and remote monitoring of patients is related primarily to the progress in such areas as IT, telecommunications and telematics [1]. The achievements in the field of telehealth presented in the literature appeared mainly in the period of the last decade [1,2,3,4,5,6].

The process of implementing telemedicine solutions to actual clinical practice has revealed new and, so far, unidentified legal, financial, technological, IT and awareness-related barriers. The highly developed countries with the greatest development of telemedicine systems have introduced and are still introducing a number of solutions facilitating the use of such systems in practice.

### 1.1. Development and Objectives of Telemedicine

In 1971, the concept of telemedicine was defined for the first time. Bird described it as ‘the practice of medicine without the usual physician-patient confrontation via an interactive audio-video communications system’ [2]. The most common definition is provided by the World Health Organization WHO, which says that ‘The delivery of healthcare services, where distance is a critical factor, by all healthcare professionals using information and communication technologies for the exchange of valid information for diagnosis, treatment and prevention of disease and injuries, research and evaluation, and for the continuing education of healthcare providers, all in the interests of advancing the health of individuals and their communities’ [7]. The main goal of telemedicine services is to ensure access to medical care for all patients, regardless of their place of residence and the possibility of moving [1,2,7,8].

Telemedicine is part of e-health, the same as information and telecommunications technologies supporting medical services [9]. For the purposes of this article, the terms telemedicine, telehealth and e-health are used interchangeably.

### 1.2. Division of Telemedicine Barriers

It is a fact that telemedicine is changing the way healthcare is delivered. Through the implementation of telemedicine services, it is possible to automatically monitor the health of a patient who stays at a distance, preventing the patient’s physical presence or when the patient’s health condition does not allow him or her to reach the doctor. Remote monitoring of a patient’s health makes it possible to improve the provision of medical services and, consequently, to improve the healthcare system. Teleconsultation, most importantly, can be of great importance in the prevention of diseases whose symptoms are underestimated or unnoticeable to patients. The advantage of implementing telemedicine is also the faster exchange of knowledge between medical professionals [7,8,10].

**Figure 1:**
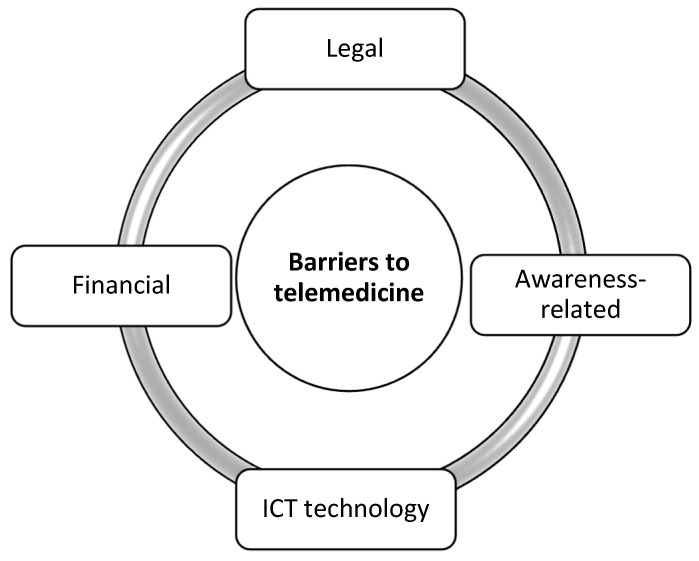
The areas of the most common barriers related to the use of telemedicine in the world [13].

Despite the undeniable advantages of such care systems and advanced technologies, the development of telemedicine systems still encounters obstacles to its effective and efficient implementation. Factors inhibiting the development of telemedicine are associated not only with patients, but also with healthcare professionals or the organisation of the medical entity itself (Figure 1) [1,11,12].

Figure 1 shows the areas where there are factors that inhibit the development of telemedicine. The biggest obstacle in implementing telemedicine solutions in the world is the belief that the costs of telemedicine are too high. Another challenge is the lack of sufficient research documenting the economic benefits and profitability of telemedicine applications, which does not encourage investment in telemedicine programmes [13,14].

Legal considerations are a significant obstacle in the implementation of telemedicine systems [13]. Issues related to the privacy and confidentiality of patient data play a key role, competing with traditional methods of delivering medical services. In addition, legal acts governing ‘virtual health services’ can be imprecise, adding to data security concerns. However, on the other hand, legal standards also affect the very organisation of health services. Unclear legal provisions regarding liability when using telemedicine practices create a huge barrier for medical professionals and the medical entity itself [1,15,16].

The awareness-related barrier also has a large impact on the implementation of telemedicine services (Figure 1). Adaptation of telemedicine systems requires the acceptance and satisfaction of users involved in the process, otherwise the implementation of telemedicine services will not be effective [17]. In the case of the awareness-related barrier, one can notice both the problem related to the opposition to adopting models other than the traditional model, but also patients’ concerns as to whether a remote medical examination will be reliable. This barrier affects mainly the elderly and people from environments with limited technological development and access to ICT systems [1,15,18,19].

Another barrier closely connected to the awareness barrier is the ICT technology barrier. Despite the fact that the use of the Internet for medical purposes has increased significantly in recent years (Figure 1), the population of elderly people who did not grow up in the era of the developing world of computers and the population of digitally excluded people may find it difficult to use such devices without proper training. The telemedicine systems used are complex and there is a potential for inaccurate handling, which may result in software and hardware failure or incorrect diagnostic readings. Not only the use of mobile devices can be a problem, but also the lack of access to the Internet. This fact is one of the possible reasons that telemedicine has not yet proved its profitability or quality improvement compared to the traditional model of providing medical services [1,15,19,20].

**Figure 2:**
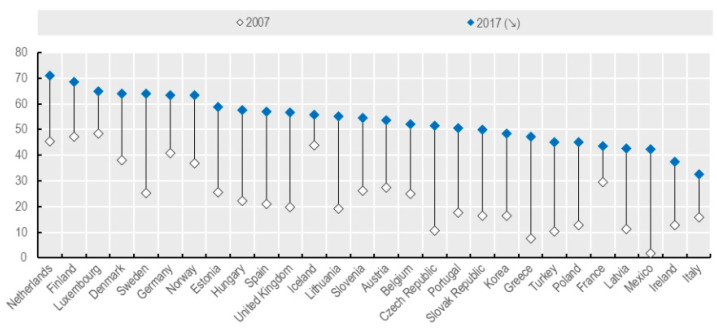
Increase in the percentage of people using the Internet for medical purposes between 2007 and 2017 [21].

The chart (Figure 2) shows increase in the number of people using the Internet for medical purposes over a period of 10 years. The recorded increase proves the growing interest in medical technologies provided remotely.

Based on the data presented above [21], people in the 25–44 age group indicated 1.5 times higher willingness to use video consultations than people over 65 years of age. However, it was assessed that the digital divide may result to a greater extent from difficult access to digital technologies than from preferences [21].

## 2. Materials and Methods

**Figure 3:**
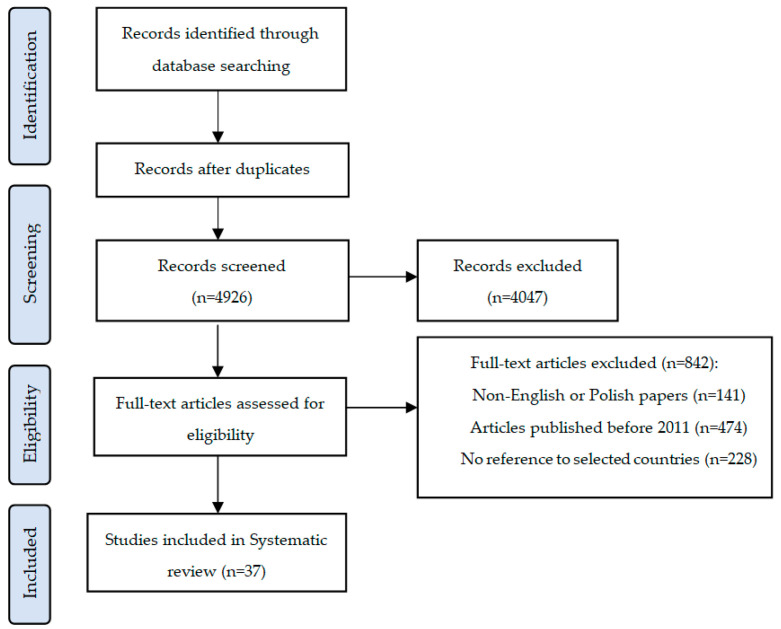
Literature selection diagram (to PRISMA).

A literature review was conducted in accordance with the PRISMA-ScR (Preferred Reporting Items for Systematic reviews and Meta-Analyses extension for Scoping Reviews) guidelines (Figure 3). In order to perform the review, the following four databases were searched: PubMed, Google Scholar, Scopus and OECD iLibrary. The search used key words such as the following: telemedicine, e-health, telehealth, telemedicine barriers, telemedicine progress, telemedicine solutions. The analysis included original scientific articles and review papers.

The literature selection qualification criteria included articles which referred to the subjects of development, barriers and telemedicine solutions in the United States and in European countries, including in Poland. The literature excluded the items which did not meet the time requirement of being published in the years 2011–2021. The time requirement results from the development of telemedicine and its current state, since the review presents solutions and problems related to the implementation of telemedicine services during the time there was a noticeable development of the technology.

## 3. Results

### 3.1. General Search Results

In total the key words resulted in 7303 search results. Duplicates were removed and analysis was performed based on the titles and abstracts of every article, in order to assess whether they qualify, and for the remaining items inclusion and exclusion criteria were applied. When creating the article, a total of 59 literature positions were used, which constituted the references.

### 3.2. Development of Telemedicine in the United States

The literature review shows that development is noticeable all over the world, but it is the United States that is recognised as a leader in the field of telemedicine. American firms have introduced many solutions to improve the operation of telemedicine and to combat the main barriers hindering its implementation [22,23].

A pioneering firm on the American market is Teladoc, whose origins date back to 2002. Its main goals were to increase access to telemedicine services, increase the quality of services, and reduce the cost of healthcare. The firm enables teleconsultation and video consultation, available 24 h. The evidence of the successive fulfilment of the intended goals is the 95% satisfaction rate among patients who received professional access to services for a lower price together with time savings [4].

In addition to firms offering patient consultations with a doctor, there are telemedicine solutions aimed at consultations between doctors. An example of such a platform is Virtual Tumor Boards, which enables the exchange of knowledge and consultations regarding the best solution in the treatment of cancer patients [24,25].

**Figure 4:**
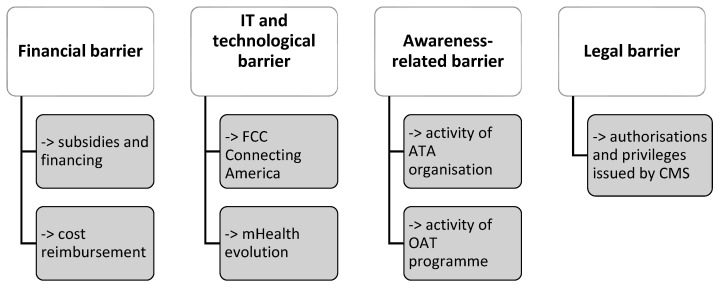
Solutions introduced in the United States to minimise barriers [24].

The data shown in the Figure 4 below indicate the areas of action taken by the United States to resolve barriers related to telemedicine so that they do not impede the implementation of telemedicine services into real clinical practice.

On the basis of the literature published so far, it can be concluded that the economic benefits resulting from the use of telemedicine services are most noticeable among patients. They are associated with the reduction in costs related to reaching the service (travel). In the context of home care, healthcare entities offering treatment with travel also see savings. As early as in 2007, around 21% of all healthcare entities dealing with home care or hospices used telemedicine services [24].

However, in the case of other types of services, the benefits of introducing telemedicine services were not visible, since it involved the purchase of equipment and its maintenance. The problem was also related to the lack of insurance. Insurance companies were reimbursing only for traditional medical services [26].

The United States, in order to minimise budgetary barriers (especially for patients), in the Medicare insurance programme and the Medicaid public health insurance programme for people with low income, established that telemedicine is not a separate form of treatment and is covered by insurance, just like traditional treatment. Additionally, patients can apply for reimbursement of any additional costs, such as technical support, fees for data transfer or equipment [24,27].

Medicare and Medicaid service centres provide support for institutions in the form of many grant programmes. The funding opportunity aims to promote both rural and urban tele-emergency services [25,28].

**Figure 5:**
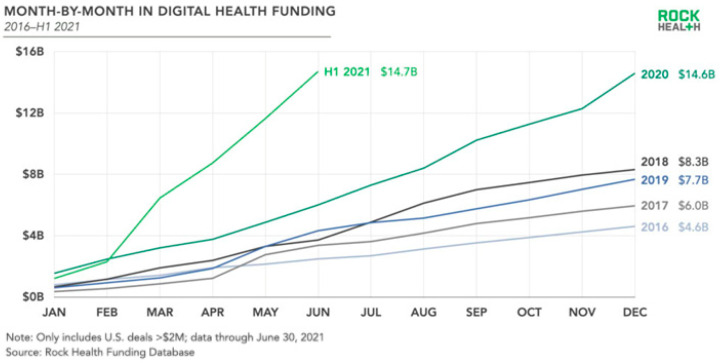
Investments in digitalisation of the health sector in 2016–2017 in the USA [30].

Figure 5 shows the increase in financial expenditure on digitalisation of medical services in 2016–2020. The chart shows the increase in funds allocated to the digitalisation of the health sector compared to previous years and the monthly financial outlay for the digitalisation of medical services in each year. On the basis of the chart, it can be seen that financing in the 2016–2020 period increased by 68% [29,30]. According to the report [31], global telehealth spending in 2021 is USD 8 trillion, of which USD 3.5 trillion in the United States.

The IT barrier is closely related to the technological barrier. The lack of broadband infrastructure necessary for trouble-free data transmission, in particular diagnostic imaging, was a challenge for the development of many forms of telemedicine. In particular, the problem concerned the ‘store and forward’ video services—based on the transmission of information via an intermediary node [29,32].

In order to expand and improve the broadband network in 2010, the United States established ‘Connecting America: The National Broadband Plan’.

The plan assumed the expansion of broadband Internet, facilitating remote monitoring of patients, support for electronic medical records. In addition, in order to close the broadband gap in cities and suburban areas, many financial initiatives have been taken to support investments in telecommunications networks. Subsidies are provided to cover the costs of building, upgrading or acquiring facilities for the purpose of providing broadband services in non-urban areas. The proposed area of services must cover at least 90% of households that do not currently have sufficient broadband access with speeds above 10 Mb/s [6,24].

The Federal Communications Commission (FCC) provides support for telehealth, whether the issue is remote patient monitoring or mobile applications accessed via smartphones, tablets, or other devices. A broadband connection enables patients to be cared for no matter where they are [33,34]. The FCC works actively with the broader healthcare system, including providers, innovators, researchers, patients and caregivers, to find ways to connect more Americans to life-saving services [34,35,36].

**Figure 6:**
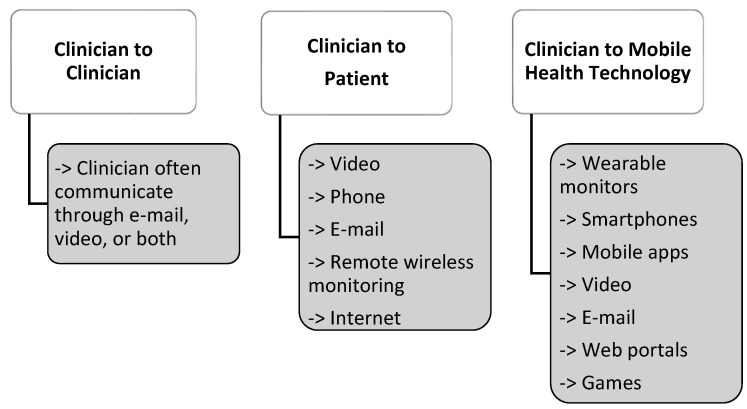
Telemedicine tools according to the American Association of Telemedicine [37].

ATA is the American Telemedicine Association, which aims to promote access to telemedicine services for both patients and healthcare professionals through telecommunications systems. The association itself describes itself as an organisation focused on accelerating the implementation of telehealth, promoting responsible social policy, and providing education and other resources to help integrate virtual care, as shown in Figure 6 [21,36,37].

Telehealth technologies, tools and services are becoming an important component of the health system. Over 60% of all healthcare facilities and 40–50% of all hospitals in the United States currently use forms of telehealth. As telemedicine tools became more common, together with an article which appeared in “The New England Journal of Medicine”, the SMART Health IT platform was launched. It allows the creation of applications that work safely in the healthcare system. The applications are linked to an electronic database and self-monitoring devices. Data are available for both patients and medical workers [37].

Another solution promoting telemedicine and combating the awareness-related barrier is the activity of the Office for the Advancement of Telehealth (OAT).

OAT provides funds to promote and improve telehealth services in ‘difficult access’ areas, including many active programmes. One such solution is the TRC (Telehealth Resource Centers) programme, which not only disseminates research results and information related to the ‘healthcare at a distance’ service. The Telehealth Resource Centers programme provides technological training for the elderly and people with difficulties in operating devices necessary to use telemedicine services [24,34,38,39].

The last discussed barrier that poses a challenge to the adoption of telemedicine is the legal barrier (Figure 4). In order to eliminate it, the Centers for Medicare and Medicaid Services (CMS) proposed nationwide authorisations and privileges for healthcare professionals. The rules for accepting telemedicine services by hospitals were introduced and the methods of granting authorisations for doctors and other workers providing telemedicine services were simplified [24,27].

The above-mentioned actions, which overcome the barriers to the widespread adoption of telemedicine, have resulted in the fact that telemedicine in the United States has reached a high level. This is evidenced by the fact that the United States is the leading country in terms of the total number of publications on telemedicine. According to Fortune Business Insights, seven out of the ten best telemedicine firms are from the United States [24,37].

### 3.3. Development of Telemedicine in Europe

**Figure 7:**
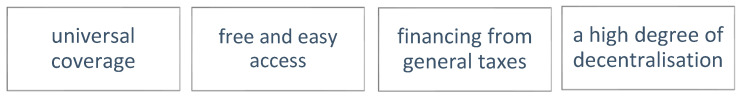
Common features of Nordic healthcare systems [42].

The development of organisational, IT and legal solutions in the field of telemedicine is also noticeable in Europe. In its reports, the European Observatory on Health Systems and Policies (EOHSP) indicated that the lowest inequalities in access to telemedicine services occur in the Nordic countries (Denmark, Norway, Iceland, Finland, Sweden). In these countries, decisions regarding the health sector have been left at the regional level, limiting central decision making. This results in increased flexibility, faster response to changes and patients’ needs, and enables adaptation to the needs of individual patient groups, as decisions are made by local authorities. All healthcare systems in the Nordic countries share four common features, as shown in Figure 7 [4,5,40,41].

In terms of digital transformation, no other region in Europe can match Denmark, Norway, Sweden, Finland and Iceland. These countries, through their emphasis on organisation and technology, create the possibility of intelligent digital solutions that support Smart Digital Health. Smart Digital Health is defined as ‘healthcare solutions that facilitate communication among healthcare professionals, between healthcare professionals and patients/clients, and solutions that enable patient/client to practise self-care’ (Figure 7). The solutions support preventive healthcare and treatment at home to avoid hospitalisation. If necessary, solutions may even support treatment at home [41].

**Figure 8:**
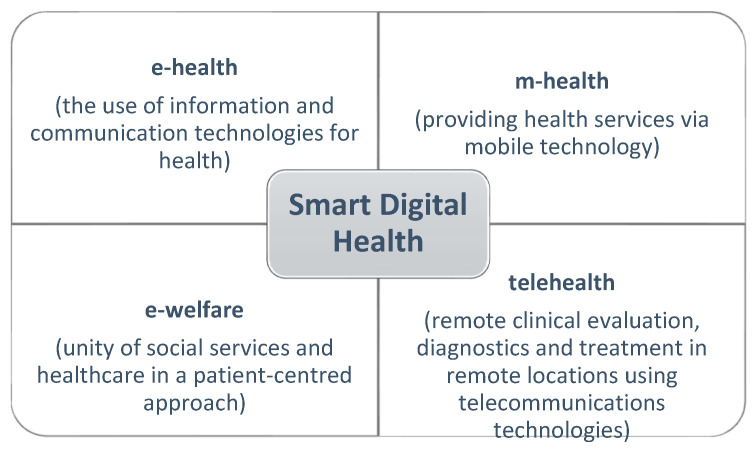
Socio-technical model for the management and provision of healthcare [42].

The figure below (Figure 8) shows the ‘Connected Health’ model that enables the provision of medical services through information technology and telematics. This model can act as a catalyst for new patient-centred procedures. Instead of ‘placing’ the patient in the system, the system becomes the environment for the patient. Therefore, the presented technological solutions in the field of medicine are collectively referred to as Smart Digital Health. They enable both the patient and healthcare system to share relevant data [41,42].

#### Tabular Review of Policies and Examples of Telemedicine Solutions in Europe

Table 1 presents the overall state of legal regulation and the current way of providing telehealth services in selected European countries where the provision of telemedicine services is allowed [43].

The digitalisation of the health sector was the basis for the development of solutions available on mobile applications. They can be used for preventive, consultative and diagnostic purposes. They enable treatment, exchange of experiences, and education of doctors through digital simulations [44]. According to data contained in [32], in the first and second quarter of 2020, over 1.2 million health monitoring applications were downloaded worldwide. This result is 34 percent higher than in 2019. Table 2 shows sample applications that are available in the European countries.

### 3.4. The State of Development of Telemedicine in Poland

Telemedicine in Poland is not defined by law. It is regulated in several legal acts. The Act of 15 April 2011 on Medical Activity presents medical activity as providing services also with the use of ICT systems or communication systems. On 12 August 2020, the regulation of the Minister of Health on the organisational standard of teleconsultations in primary healthcare was issued [45].

Compared to other countries where telemedicine services are used, Poland is a novice in the field of telemedicine. The report published at the turn of 2016 and 2017 indicated that only 7% of the population (out of 38.5 million) uses medical services via the Internet. The average in the European Union countries is 13%. The report indicates that 98% of the population has no concerns about e-privacy, and 48% of the population seeks information about their health via the Internet. Polish patients have a tendency to use digital services. The desire for medical visits with appointments made by the Internet was expressed by 90% of the patients. The report has emphasised that persons aged above 60 have also indicated a positive impact of the digitisation of the health care sector. The data show that it is not the awareness-related barrier that determines the low rate of interest in using telemedicine services in Poland [46,47,48].

In a comparative summary of the Visegrád Group countries [49], Slovakia performed above EU average concerning the above basic digital skills of its citizens. In 2017, in particular in Poland, only 20% of population had above basic overall digital skills. Almost 25% of citizens of Poland, Czechia and Hungary had low or no overall digital skills. This result corresponds to a low indicator of the use of services, also medical services, through Internet technologies [49].

In the Digital Economy and Society Index (DESI) for the year 2020, Poland was in 23rd position out of 28 countries of the European Union [50]. The state of development of telemedicine both in Poland and in other countries may result from the IT infrastructure. It may form a barrier for the digitisation of the medical sector. The development of this infrastructure increases the possibility of using telemedicine solutions and has an impact on the intensive increase in telemedicine services [51,52].

**Figure 9:**
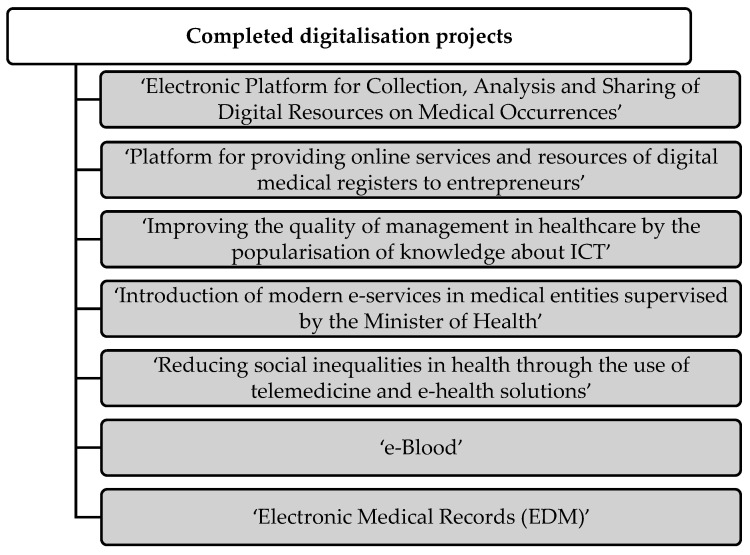
Existing solutions for digital transformation of the health sector [44].

**Figure 10:**
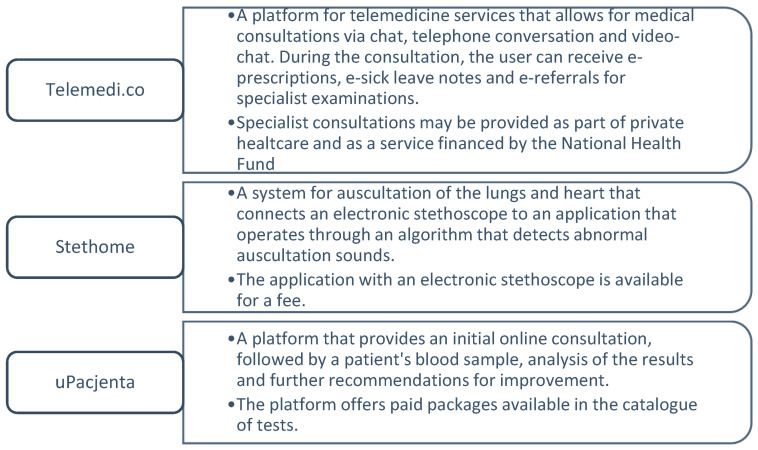
Examples of Polish startups as successfully implemented telemedicine solutions [53].

In order to develop digitalisation of the health sector, projects were adopted and successively implemented (Figure 9) [44], and also platforms and technological solutions appeared on the market, examples of which are presented in Figure 10 [53].

The ‘Electronic Platform for Collection, Analysis and Sharing of Digital Resources on Medical Occurrences’ project provided the possibility of issuing and using e-prescriptions, e-referrals, and introduced the Patient Internet Account (IKP). By setting up an account with IKP and then logging in, patients have access to their medical records. Patients also have the option to change their doctor or primary care nurse. The implementation of the project allowed for access to medical records during treatment abroad, in Europe, through cooperation with European electronic platforms [44].

Through the second project, ‘Platform for providing online services and resources of digital medical registers to entrepreneurs’, the Medical Registers Platform was established, as well as the Document Exchange System. The integration of these two projects ensures a more efficient exchange of documents and also fast and constant access to data [44].

‘Improving the quality of management in healthcare by the popularisation of knowledge about ICT’ is an educational project completed in 2015, consisting of 24 regular meetings attended by medical workers and management staff. The project was aimed at employees who were unable to use IT systems in their workplace. During the meetings, knowledge on the use of modern technologies in the workplace was shared [44].

‘Introduction of modern e-services in medical entities supervised by the Minister of Health’ is an e-service project that will end in 2022. It assumes the implementation of e-services in selected medical facilities, including not only electronic medical documentation, but also e-prescriptions, e-registration and e-analyses [44].

The aim of the ‘Reducing social inequalities in health through the use of telemedicine and e-health solutions’ telemedicine project is to reduce the costs of medical procedures and facilitate access to medical services for patients. The project covers several areas of medicine: cardiology, geriatrics, obstetrics, palliative care, chronic diseases, diabetes and psychiatry [44].

The ‘e-Blood’ project provides information exchange between blood centres and blood donors. Blood donors can remotely arrange an appointment at a blood collection facility and check the test results or the donation calculator. Healthcare entities gain access to remote blood ordering [44].

‘Electronic Medical Records (EDM)’ involve the digitalisation of medical records in accordance with the CDA HL 7 standard. EDM include information on diagnosis, results, recommendations or refusals of admission to hospital, e-prescriptions and e-referrals, as well as an information card covering the entire process of inpatient treatment [44].

In order to disseminate telemedicine services, the private e-health sector has introduced many new solutions. The Top Disruptors in Healthcare report, published in 2021, presents innovative medical startups in Poland that use advanced computer technologies and artificial intelligence algorithms. This is the second edition showing Polish medical innovations. The report emphasises the need to create conditions and eliminate barriers so that medical projects can be successfully implemented in Poland as one of the leaders of the Central European region in the area of startups. The report included 115 startups, 41 more than in the previous edition in 2020. As many as 63 startups indicated telemedicine as their area of activity [53].

During the multiple-choice questionnaire survey, the vast majority, as many as 62%, chose financing from the founders’ funds as a source of financing. This is a significant increase compared to the previous year, in which only 31% of the surveyed startups financed themselves from their own funds. The second most common answer of the surveyed startups was funding from European grants—34% and domestic grants—31% [53].

The Polish healthcare system is largely financed from public funds. However, as listed in Figure 10, telemedicine solutions indicate the necessity to make payments in order to use them. The public payer’s funds are used to finance oncological, cardiological and geriatric tele-case conferencing as well as hybrid cardiological rehabilitation [50,53].

Tele-case conferences are a remote consultation of medical specialists with each other and with patients. They are settled by the healthcare provider participating in the tele-case conference. A cardiological tele-case conference includes an interview, analysis and interpretation of test results as well as recommendations and determination of further treatment stages. A geriatric tele-case conference is offered to patients over 65 years of age and also includes an interview, analysis of test results, analysis of pharmacological treatment and further recommendations [54].

As part of comprehensive care after myocardial infarction (KOS-Cardiac infarction), the KOS regulation enabled remote cardiac rehabilitation in the patient’s home conditions. Hybrid cardiac rehabilitation consists of a short rehabilitation in a hospital ward, followed by telerehabilitation with the use of a device monitoring rehabilitation sessions at home [54,55].

In addition, in 2021, the National Health Fund launched the First Contact Teleplatform. It is one toll-free number for citizens of the entire country. Advice is provided not only in Polish, but also in English, Ukrainian, Russian, as well as in sign language via video chat and by a Polish Sign Language interpreter. The services of the First Contact Teleplatform can be used outside the working hours of primary healthcare [56].

## 4. Discussion

When analysing the current state of development of telemedicine in Poland, it is necessary to present the four main challenges of digitalisation of the health sector, which were presented in the report of 2021 entitled Digital Health [44].

Ensuring the security of data, in particular medical data, is an important issue for patients who use devices or sensors for monitoring health parameters that are connected with applications. Along with the spread of applications and telemedicine platforms, the amount of processed and collected data increases [44].

Another challenge is to improve Internet access. According to the data for 2020, collected by Statistics Poland, nearly 4 million Poles (aged 16–74) have never used the Internet, and 85% of them are people over 55. The lack of access to the Internet, but also the inability to use it, contributes to the inhibition of the development of telemedicine services. Additionally, it is the people who have difficulties with adapting modern telemedicine solutions that constitute the group of the greatest beneficiaries of this form of access to health services [44].

An important step in the digitalisation of the health sector is to provide a system of reimbursement/co-financing of technological solutions based on personal devices communicating with specialised applications. The creation of the Electronic Medical Records (EDM) and the Patient Internet Account (IKP) is the beginning of the development of digital medical services in Poland [44].

The fourth challenge is to design a fair system of assigning responsibility. AI-powered applications and devices challenge standard clinical practices of moral responsibility and security. The responsibility dilemma was described in [55,56] on the basis of the AI Clinician system developed by scientists from Imperial College London. The system supports decision making in the treatment of sepsis. The system, by analysing data from the electronic patient register, sends personalised recommendations every 4 h. When making decisions, the doctor relies on the recommendations obtained by the artificial intelligence system. The dilemma of whether a doctor, system creator or system security engineers bear moral responsibility in the event of a mistake leads to the creation of a precise regulatory framework [57,58,59].

In order to meet the above challenges, the following authors’ recommendations together with explanations which may contribute to the development of telemedicine services in Poland are listed below:

1. To ensure consistency in the provisions regulating the scope of admissibility of providing telemedicine services for medical professions.
  The Act on Medical Activity indicates the possibility of providing medical services with the use of telemedicine solutions. However, in some acts regulating the medical professions there are no provisions allowing for the provision of telemedicine services. This is the case with acts on the professions of a physiotherapist, laboratory diagnostician and psychologist as well as the Act on the State Medical Rescue. Moreover, a general provision in the acts should indicate the possibility of providing services in a telemedical way, when it is beneficial for the patient [45].
2. Implementation of the process/procedures for evaluating services using artificial intelligence.
  The procedures will minimise errors in automated decision support systems, and also will allow for error correction at an early stage. The implementation of audits by minimising errors will also reduce the problem of assigning responsibility for these errors.
3. To introduce a series of educational training sessions for citizens on the use of digital solutions.
  A series of training session for people affected by digital exclusion in terms of benefits, Internet security, but also the practical use of digital solutions. Training sessions would take place cyclically in many regions of Poland in order to provide educational support to as many citizens as possible.
4. Reimbursement/co-financing of solutions used in telemedicine services.
  In order to reduce the phenomenon of digital exclusion, access to medical devices and devices used for telemedicine purposes should be improved by covering them with reimbursement. An example of a solution could be a ‘prescription application’. Once selected from the catalogue, the application would be verified and prescribed by a physician and would be reimbursable [44].
5. Providing financial support for new, developing enterprises related to telemedicine.
  Many private enterprises choose to start their activities in the use of technology in the medical industry. Their implementation and development require a high investment outlay. Providing financial support for the start of telemedicine activities and assistance in its development would provide benefits not only for entrepreneurs, but also for the entire healthcare sector.
6. Creation of one superior telemedicine system.
  A telemedicine system collecting data from all telemedicine applications, combining e-documentation of the patient, including the following: prescriptions, medications taken, received doses of ionising radiation, authorisations, and access to which would be available to healthcare providers throughout the country. The healthcare provider would only authorise medical workers who would participate in the patient’s treatment to access the documentation. The establishment of such a system would guarantee more effective treatment and more efficient assistance, especially in the case of people with whom contact is difficult.

Recommendations of the authors, directed at the increase in the use of telemedicine solutions apply to many countries across the world. They are intended mainly for health care decision makers and regulators, in particular for the Minister for Health or for the payer/payers (since it is them who create the framework of the health care system in every country). According to the authors, only the use of telemedicine solutions will allow us to solve the issue of the shortage of medical professionals (in particular doctors).

## 5. Conclusions

The emergence of new opportunities related to telehealth and their integration with IT systems make it possible to create healthcare based on equal and increased access to medical services.

The development of digitalisation of the health sector, both in Poland and in the world, is constantly limited by a number of barriers, which were discussed in the article. In order to fully use the potential of telemedicine, one should start with activities enabling the minimisation of these barriers. Based on the experience from countries where the implementation of telemedicine services is at a higher stage, the Polish healthcare system could develop such a model of activities that would not have any gaps and legal inconsistencies. It would ensure the education of people from the digitally excluded environment so that potential patients are fully trained in the available, reimbursed solutions.

Despite many barriers to the digitalisation of the Polish health sector, the Polish market is full of startups. The results of the survey on financing startups, which show a significant predominance of financing from the resources of the founders, may indicate both difficulties in obtaining funds from external sources, but also taking into account the second most common answer—grants, greater awareness and faith in the development of telemedicine solutions among founders.

The collected material shows that the development of digital technologies offers a wide range of possibilities, the use of which can improve the provision and use of healthcare services. The discussed topic of the article does not exhaust all the issues related to telemedicine, barriers which it still faces, methods of solving these problems and its proper implementation.

## Figures and Tables

**Table 1:** State of legal regulations of telehealth services and examples of telemedicine solutions in selected European countries [43].

Country	Policy and Telemedicine Solutions
Croatia	1. Telehealth (or ‘Telemedicine’) is defined in the Health Protection Act as the provision of healthcare services at a distance using information and communications technologies. 2. Telemedicine in Croatia covers such fields of medicine as follows: cardiology, radiology, neurology, family medicine, pulmonology, neurosurgery, and emergency medicine. The authorities support the development of telemedicine by providing the Ministry of Health with the equipment necessary to work in telemedicine centres.
Denmark	1. There are no specific rules on telehealth. It is regulated by legal acts related to health. 2. The development and implementation of telehealth services was of the utmost importance. To this end, the Danish authorities developed digital solutions in the form of a digital platform called ‘sundhed.dk’ and ‘Min læge’ or ‘Medicinkortet’ applications. The platform together with the applications enables access to a family doctor and also enables to renew prescriptions. It also has an option to remind the user to take medication. The use of the above services is free.
Finland	1. The Finnish National Supervisory Authority for Welfare and Health (‘Valvira’) authorises the provision of telemedicine services for the purpose of clinical consultations, diagnostics, monitoring, treatment and making all clinical decisions. The Ministry of Social Affairs and Health confirmed that in terms of content, telemedicine services do not differ from traditional medical services. 2. Primary healthcare is provided by local health centres. Specialised medical care is provided by district hospitals. Moreover, in some cities, e.g., in Helsinki, it is possible to book a visit to Health Stations in order to assess the condition of the skin. A national medical helpline was launched, through which healthcare workers provide psychiatric care to patients. The costs of telemedicine care are usually covered by the municipality to which the patient belongs. The fees paid for the services provided are governed by the regulation on the fees for services related to customer service. The National Health Insurance scheme also covers fees for using private medical services.
France	1. Telemedicine services are regulated in the Public Health Code, which was introduced in 2008 and is updated by decrees of the French parliament. Telemedicine services can be provided by authorised medical professionals, regardless of whether the medical facility is a public or private facility. 2. The use of telemedicine services is reimbursed in the same way as the traditional method of provision, if the specified path is followed. The physician in charge must refer the patient for a teleconsultation. A teleconsultation can only be provided by a physician who knows the patient and who consulted the patient at least once in a traditional way, not earlier than 12 months before.
Greece	1. The Act allows for services under the responsibility of a doctor who deals with a specific case. The patient signs a consent to use telehealth services, and if this is not possible, consent is obtained from the first degree relative. 2. Telehealth services are provided through the National Telemedicine Network. Its activity began in 2016. Its mission is to ensure access to health services for the inhabitants of remote Aegean islands to ensure that they have constant access to care despite geographical limitations.
The Netherlands	1. Telehealth is allowed in the Netherlands and is part of a stimulus package to develop innovation in healthcare. There are no specific regulations for telehealth, it is considered an integral part of healthcare. 2. There are many telemedicine solutions. Among them is health monitoring, providing support to people with mental illnesses, tele- and video consultations of medical workers. The Dutch Healthcare Authority provides reimbursement for online consultations. In addition, in individual regions, an e-health week is organised, during which telemedicine solutions are promoted.
Ireland	1. Telehealth services are regulated in the Health Act. Healthcare workers providing this type of services work under applicable and updated regulations and a code for the performance of a specific profession. To a large extent, legal regulations cover the problem of cybersecurity and data protection. 2. There is no limit to the services that can be provided remotely. The Telehealth Committee included Microsoft Teams, Skype for Business, Cisco WebEx and, in exceptional cases, WhatsApp in its telemedicine solutions. Telemedicine services are provided by state agencies and private clinics.
Germany	1. In Germany, telehealth requirements are not regulated by a single legal act, but by many different acts, regulations and directives. Aspects relating to remote treatment, prescriptions, reimbursement, or requirements for documentation and informed consent are regulated, inter alia, in Model Professional Code for Physicians, Social Code or the Medicinal Products Act. 2. Telemedicine can be an integral part of almost any medical specialisation. Telehealth applications and technologies must be approved by the German federal authorities. Telehealth applications/technologies that are currently authorised in Germany include online consultation, remote diagnostics and monitoring of, for example, patients with cardiac resynchronisation therapy (‘CRT’) implants, with implantable cardioverter-defibrillators (‘ICDs’). Teleconferencing applications and platforms such as Skype, Zoom, etc. are not permitted to be used to provide telemedicine services.
Portugal	1. The use of telehealth services is regulated in accordance with the principles of medical ethics as well as decisions and standards issued by the National Health Service. 2. Telemedicine is used in all medical areas. Regional authorities provide access to equipment necessary for the provision of, for example, teleconsultations. The National Health Service defines the format for the delivery of e-health services, both real-time and deferred in the form of data storage and transmission. The first consultation with a doctor takes place in the traditional way. Only subsequent visits can be carried out remotely.
Slovenia	1. Slovenian law recognises and defines two types of telehealth services. It is ‘telemedicine’ and ‘telepharmacy’ (a way of providing advice at a distance through modern telecommunications technologies involved in pharmaceutical activities). 2. Telehealth services are focused largely on monitoring the health of patients with diabetes and heart disease. Patients use specialised monitoring devices, and information is transmitted to the Health Centre via mobile devices. Patients and healthcare professionals have access to the health information system operating at the national level. It enables to issue prescriptions, book medical appointments, and access your medical data.
Hungary	1. Telehealth services are allowed within the framework of healthcare services in Hungary. However, there are healthcare legal acts that apply to telemedicine and set minimum requirements for telemedicine services to be delivered remotely. 2. Healthcare professionals are authorised to provide advice, diagnose, conduct consultations, issue a referral, conduct therapy and rehabilitation classes, issue prescriptions. The condition for providing telemedicine services is a reasonable justification given by the patient, but also a medical worker, and a technological requirement, limited only to devices enabling video conversation and chat. A patient who uses this type of healthcare receives a leaflet on telehealth services. The provider of telemedicine services is responsible for broadband Internet access, transmission stability and data security.
United Kingdom	1. Telemedicine services are not regulated in a specific act. Professionals who provide this type of medical service must follow the guidelines to ensure the quality of treatment. For example, the General Medical Council issued criteria for remote consultation and the General Pharmaceutical Council established guidelines for online pharmacies. 2. Provision of telemedicine services includes consultations, diagnoses and remote treatment. Psychological support is provided through telephone and video calls. There are numerous applications available that offer a range of telemedicine services. The UK National Health Service developed a specific Technology Enabled Care Services (TECS) programme. The programme was created to increase patients’ awareness of the benefits of telemedicine services.
Italy	1. Telehealth in Italy is regulated by provisions on traditional health services. The guidelines of the Ministry of Health define telemedicine services not as a replacement, but only as a tool supporting the traditional model of treatment. 2. Service Centre is established to manage the data exchanged between the patient and the healthcare provider. The guidelines of the Ministry of Health define telemedicine as a tool supporting, in particular, secondary prevention, when the patient is at risk or is already diagnosed, e.g., a diabetic, a person with cardiovascular diseases, but also as a tool facilitating patient monitoring.

**Table 2:** Review of sample digital solutions [44].

Purpose of the Applied Digital Solution	Sample Digital Solutions	
Prevention	** 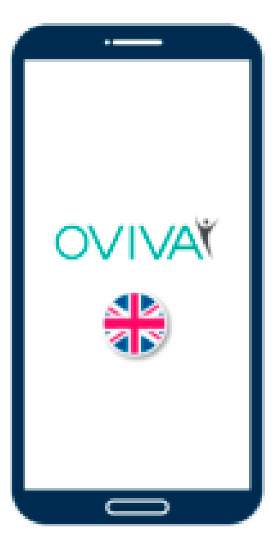 **	The United Kingdom uses the services of the ‘Oviva’ technology. The application supports people who require a specialised health diet. Through the application, the patient contacts a specialist who adjusts his nutritional programme. The patient can monitor his or her progress as well as constantly contact a specialist. The application also allows for education on healthy eating.
	** 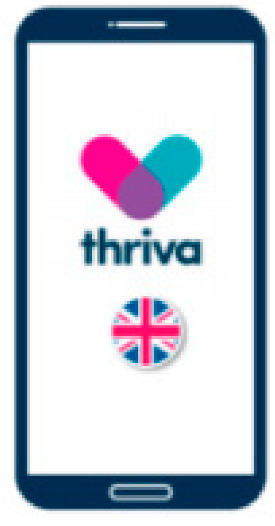 **	The ‘Thriva’ application is used by UK citizens. It aims to improve health outcomes through patient self-control. The patient uses a home kit to perform a blood test and enter the results into the application. Then, the patient obtains a personalised plan to obtain the correct level of cholesterol, micronutrients in the blood, prevent liver disease or diabetes.
Consultation	** 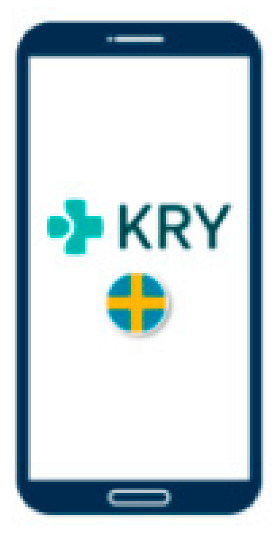 **	The ‘Kry’ application is used in Sweden. Doctors, nurses and psychologists are registered in the application. The patient makes an online reservation. Then, the qualified worker connects with the patient at the appointed time. If necessary, the worker prescribes medications or refer the patient for further consultation. The application enables to book a visit to a specialist for a physical examination. ‘Kry’ provides constant access to medical records.
	** 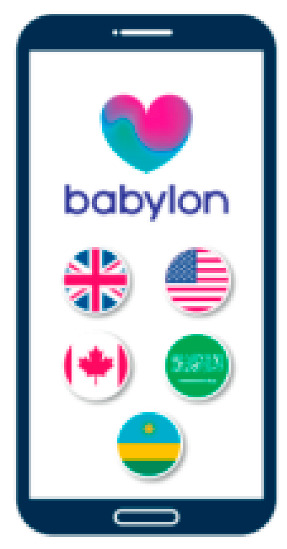 **	The ‘Babylon’ application available in the UK. Outside Europe, it is available in the USA, Canada, Rwanda and Saudi Arabia. The provider of the application is the British Health System. The application allows fora remote consultation with medical staff. Additionally, due to the use of artificial intelligence, it relieves the staff of their duties. The artificial intelligence system reads and learns from anonymised medical data sets, if the patient consents to the use of data about his or her health. The artificial intelligence system helps the doctor determine the cause of the patient’s symptoms, but also make a prognosis of the patient’s health.
Diagnosis	** 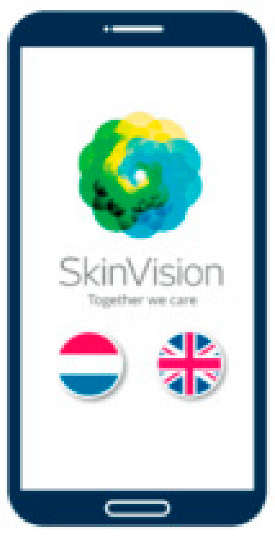 **	‘Skin Vision’ is an application used in the Netherlands and the United Kingdom since April 2021. The application is also available to users in Poland. Based on the photos, the application creates a map of birthmarks, monitors them and analyses the risk of skin cancer development. The user is given a recommendation on what steps he or she should take depending on the results obtained. The application combines artificial intelligence with the knowledge of specialists.
	** 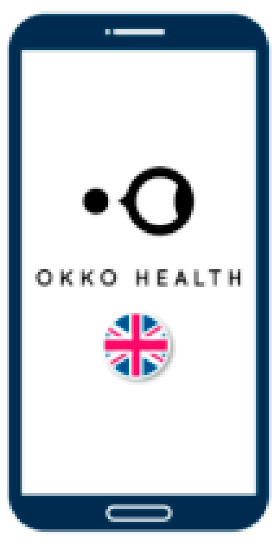 **	The ‘OkkoHealth’ application, which is used in the United Kingdom, enables patients to diagnose their eyesight. Through remote examinations (visual acuity, contrast or colour vision) it monitors and predicts the development of the disease. Examination data are stored and analysed by specialists who contact patients via the application.
Treatment	** 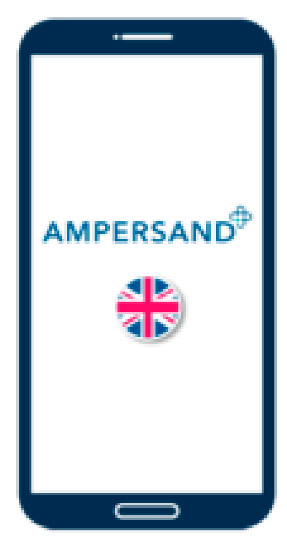 **	‘Ampersand’ is a British application used by hospitals for people suffering from ulcerative colitis, arthritis and patients with inflammation. The application provides access to expert-led courses on health improvement. It enables to track symptoms, habits, activity and diet, and sends a weekly general health report to the user.
	** 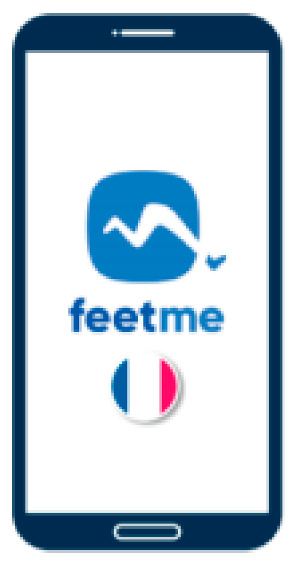 **	‘Feetme’ is an application available in France. Through special insoles integrated with the application, it helps to improve the quality of gait in people during rehabilitation or after illnesses. The application collects data in real time, and the stimulation with the use of smart insoles helps the patient to maintain proper gait.
Education	** 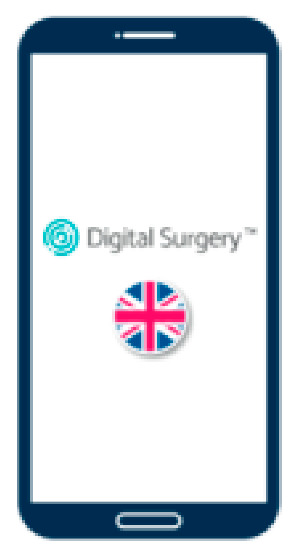 **	‘Digital Surgery’ cloud solution being used in the United Kingdom, stores video recordings of performed surgical procedures. Additionally, the user can attach his or her own notes to each recording and share them with other users. Moreover, ‘Digital Surgery’ provides a library of interactive simulations for learning purposes.
	** 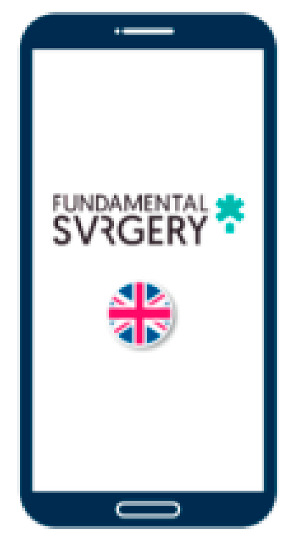 **	‘Fundamental Surgery’ is a virtual reality platform which is used in hospitals in the United Kingdom. It is used as a training tool. It provides sound, view and touch feeling when simulating surgical operations.

## Data Availability

The data presented in this study are available on request from the corresponding author.
